# Privacy-aware and Efficient Student Clustering for Sport Training with Hash in Cloud Environment

**DOI:** 10.1186/s13677-022-00325-2

**Published:** 2022-09-28

**Authors:** Guoyan Diao, Fang Liu, Zhikai Zuo, Mohammad Kazem Moghimi

**Affiliations:** 1 College of Physical Education, Southwest Forestry University, Kunming, China; 2 Faculty of Foreign Languages, Southwest Forestry University, Kunming, China; 3 Department of Communication Engineering, University of Sistan and Baluchestan, Zahedan, Iran

**Keywords:** Cloud platform, User clustering, Privacy, Efficiency, Local server, Hash

## Abstract

With the wide adoption of health and sport concepts in human society, how to effectively analyze the personalized sports preferences of students based on past sports training records has become a crucial and emergent task with positive research significance. However, the past sports training records of students are often accumulated with time and stored in a central cloud platform and therefore, the data volume is too large to be processed with quick response. In addition, the past sports training records of students often contain certain sensitive information, which probably discloses partial user privacy if we cannot protect the data well. Considering these two challenges, a privacy-aware and efficient student clustering approach, named PESC is proposed, which is based on a hash technique and deployed on a central cloud platform connecting multiple local servers. Concretely, in the cloud platform, each student is firstly assigned an index based on the past sports training records stored in a local server, through a uniform hash mapping operation. Then similar students are clustered and registered in the cloud platform based on the students’ respective sport indexes. At last, we infer the personalized sport preferences of each student based on their belonged clusters. To prove the feasibility of PESC, we provide a case study and a set of experiments deployed on a time-aware dataset.

## Introduction

With the wide spread of COVID-19 all over the world, people are more focusing on health and life than ever before [1–3]. In this situation, health and sport concepts have gained wider adoption in whole human society than ever before [4–6]. To achieve the goal of healthy living and life, sport courses have been playing an increasingly important role in the whole education system. As a precondition of sport course setting and optimization, accurate recognition of personalized sport preference of each student is becoming a crucial and emergent task in front of education systems. Fortunately, past sports training records of students have provided a theoretically feasible evaluation basis to cluster the students and then infer their personalized sports preferences accordingly.

However, the past sports training records of students are often accumulated and stored in a central cloud platform for years and as a result, the data volume is often big enough [7], which probably leads to a long response time in student clustering and subsequent sports preference identification. In addition, past sports training records often contain some sensitive user information of students, which probably discloses student privacy if we cannot protect the data transmitted to the cloud platform well [8–10]. Considering these two challenges, a privacy-aware and efficient student clustering approach, named PESC is proposed for sport preference discovery and mining in cloud environment.

Concretely, in the proposed PESC approach, each student is firstly assigned a sport index (with less private information) by analyzing his or her past sports training records registered in the cloud platform through hash projection operations. Secondly, similar students are clustered into different groups based on students’ less-sensitive sport indexes recorded in the cloud platform (since index-based similarity calculation is rather quick, we can guarantee a high clustering efficiency in cloud). Third, we determine the personalized sport preferences of each student based on their belonged groups or clusters. At last, we demonstrate the effectiveness and efficiency of PESC through an intuitive example constructed from real world and a set of simulation experiments.

The major contributions of this article are detailed as below.

1. Past sports training records as well as their respective time tags are used as a feasible and promising evaluation basis to infer the personalized sport item preferences of students in cloud-enabled smart education.
2. Hash index mechanism is recruited here to cluster the students into different groups based on the past sports training records registered in the cloud platform. In concrete, firstly, we convert the sensitive user data into a less-sensitive user index through a kind of hash mapping process. Secondly, we use the less-sensitive user indexes to cluster users into different groups without disclosing much user privacy. This way, we can guarantee the user privacy is secure during the accurate user clustering process.
3. We present a case study extracted from real world applicable scenarios to demonstrate the detailed steps of the proposed PESC solution. In addition, a set of simulated experiments are also provided to show the feasibility of the proposed PESC solution.

The rest of paper is briefly introduced as follows. Literature investigation is conducted in Related literature Section. A motivating example Section clarifies the research background and significance of this paper with a vivid example. Our solution: PESC Section introduces the detailed student clustering process as well as the student sport preference recognition process. A case study is presented in Case study Section and evaluation is presented in Evaluation Section. We summarize the paper and point out the possible research directions in Conclusions Section.

## Related literature

In this section, we investigate the current research associated with sport clustering in cloud computing.

In [11], the authors mainly discuss how the socio-economic proximity between organizations or individuals affects the development of sports clusters. In order to solve this problem, the authors mainly investigate the sports groups of surfing and sailing, and puts forward a two-step model of cluster development. The purpose of [12] is to explore whether and how sports clusters correlate with community resilience across regions. To answer this question, the authors apply geographically weighted regression and visualization techniques to the macro data detection of community resilience. The results show that the community resilience of sports industry cluster is significantly correlated with the community resilience, and the two are strongly positively correlated. In [13], the purpose is to use the Sports eFANgelism scale to classify the fans in the process of The Korean league and analyze the differences between groups. Through cluster analysis, three groups were identified according to the level of league fans’ behavior. At the same time, through variance analysis, each group had obvious differences in four kinds of preaching behaviors. In recent years, the holding of a large regional event is considered to have a potentially positive impact on the region’s economy [14]. Concretely, the authors attempt to delve deeper into this field, focusing on the impact of participatory events on participants themselves. The authors use a cluster analysis procedure to perform a combinatorial analysis of participants, thus confirming and discussing the existence of various effects associated with participation events.

In [15], the authors mainly survey and give feedback to professional athletes. In this study, professional identity, sports identity and self-efficacy are measured, and cluster analysis is used to analyze the survey results. It is proved that identity and self-efficacy are important factors for athletes to choose dual career path. In [16], the aim was to replicate a controlled cluster experiment that demonstrated that behavioral skill training significantly improved motor behavior and self-efficacy in adolescents. Results from repeated trials show heterogeneity in the effectiveness of sports-based interventions, even among apparently similar populations. In [17], physical education has become an important course, and physical education has become an important teaching mode. This paper mainly studies the influence of physical education on college students’ physical quality and physical activity level. In this study, the authors used a cluster randomized trial to verify. The results show that under the environment of limited control and self-evolution, appropriate physical education has a positive effect on the development of college students’ physical quality. Nowadays, many young athletes are found to be taking performance-enhancing drugs [18]. In order to more accurately understand the causes of adolescent doping, this paper verified the Sports Drug Control model of Adolescent athletes (SDCM-AA), and modified the SDCM-AA model according to the experimental situation. The results show that there is a close relationship between the doping situation of athletes and the control mode of sports drugs, the cluster effect and the value of athletes’ own norms.

With the above literature review, we can simply conclude that existing literatures about population clustering based on sports information often fall short in time efficiency and privacy protection especially in the big data context. Considering this limitation, we propose a highly efficient student clustering approach with privacy protection, named PESC in this paper.

## A motivating example

**Fig. 1:**
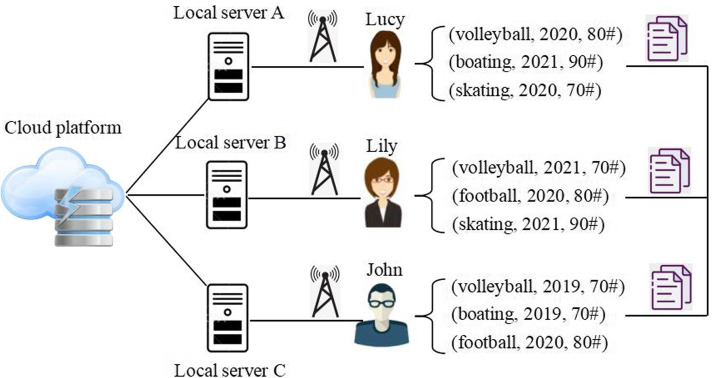
An integration framework of sport-time-score records in cloud environment

As shown in Fig. 1, three students (i.e., Lucy, Lily and John) as well as their past sport score records are presented and stored in local servers A, B and C, respectively. For example, Lucy took three kinds of sport items (i.e., volleyball, boating and skating) in the past and her volleyball score in 2020 is 80, boating score in 2021 is 90 and skating score in 2020 is 70. Likewise, the scores of the other two students are also presented in Fig. 1. For uniform data analysis and mining, the scores of students stored in different local servers A, B and C need to be transmitted to a remote cloud platform.

As mentioned in Fig. 1, different users’ sport-time-score records are stored in different local servers and finally transmitted to a central cloud platform for uniform processing. During such a data transmission process, user privacy contained in the users’ sport-time-score records are probably disclosed to other parties. With the known sport-time-score records of students in the cloud platform shown in Fig. 1, we can divide the students into different groups and infer their respective sport preferences for better sport training. However, two challenges often exist in the above student clustering and sport preference recognition process. First, too many students (although only three students are exampled in Fig. 1, the student volume is often big enough in practice) as well as their respective sport score records are involved in uniform data analysis in cloud platform, which often consumes much computational time and leads to a longer waiting time (specifically, the accumulated sport-time-score records of students are continuously growing with time elapsing, which often calls for more processing time). Second, the sport-time-score records of students often contain partial privacy of the involved students and therefore, they are often reluctant to publish their sensitive sport-time-score records to the public.

Motivated by the above two challenges, a privacy-aware and efficient student clustering approach, named PESC is proposed here for achieving uniform data analysis and mining in cloud environment. The concrete steps of PESC will be clarified with more details in the following sections.

## Our solution: PESC

**Fig. 2:**
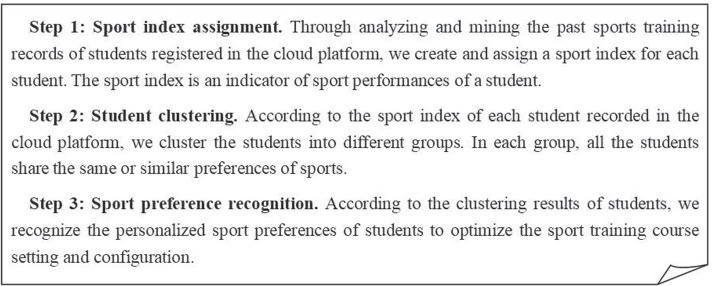
Detailed procedure of PESC

Next, we briefly introduce the major steps of the proposed PESC solution: firstly, we convert the sensitive user data into a less-sensitive user index through a kind of hash mapping process; secondly, we use the less-sensitive user indexes to cluster users into different groups without disclosing much user privacy; third, we determine the personalized sport preferences of each student based on their respective belonged groups or clusters. Concrete description of the PESC solution can be found in Fig. 2.

### Step 1: Sport index assignment

In this step, we create and assign a sport index to each student based on his or her sport score records in the past registered in the cloud platform. Here, the index is created by a kind of hashing technique [19–21], whose reason is as follows: distributed data integration from mobile clients to cloud platform is often not secure [22–26] while hashing has been proven an effective data protection technology. Please note that the sport score records are time-aware as exampled in Fig. 1. Therefore, to smooth the subsequent sport index creation and assignment procedure, we first model the time-aware sport score records with the sport-time-score matrix *M* in Eqs. (1)-(2). Here, we assume there are *n* students, i.e., $Stu\_set$ = $\{s_{1}, s_{2},\ldots , s_{n}\}$, *m* kinds of sport items, i.e., $SI\_set = \{ si_{1},\ldots , si_{m} \}$ and *l* time slots, i.e., $T\_set = \{ t_{1},\ldots , t_{l} \}$. Thus, each column in matrix *M* in Eq. (1) denotes a student in $Stu\_set$, each row of $s_i$ in Eq. (2) indicates a time slot and each column of $s_i$ in Eq. (2) represents a sport item. With the above formulation, we can find that each entry $q_{i,j,k}$ $( 1 \le i \le n, 1 \le j \le m, 1 \le k \le l )$ in Eq. (2) means student $s_{i}'s$ score of sport item $si_j$ at time slot $t_k$, which has been exampled in Fig. 1.

1 $$\begin{aligned} M=\left( s_{1}, s_{2}, \ldots , s_{n}\right) \end{aligned}$$

2 $$\begin{aligned} s_{i}=\left[ \begin{array}{ccc} q_{i, 1,1} & \cdots & q_{i, m, 1} \\ \vdots & \ddots & \vdots \\ q_{i, 1, l} & \cdots & q_{i, m, l} \end{array}\right] \end{aligned}$$

Specifically, if student $s_i$ has not taken sport item $si_{j}$ at time slot $t_k$, then the corresponding entry $q_{i,j,k}$ in Eq. (2) is equal to zero, i.e., $q_{i,j,k}= 0$ holds. Next, we create and assign a sport index $Y_i$ for each student $s_{i} ( 1 \le i \le n)$ in matrix *M*. Concretely, we convert the matrix $s_{i}$ in Eq. (2) into a corresponding vector $v(s_i)$ which is formalized in Eq. (3). Please note that as Eq. (3) indicates, $v(s_{i})$ is an l*m-dimensional vector. For brief formalization, we assume $l*m = Q$ in the following discussions. Thus, $v(s_i)$ is a *Q*-dimensional vector.

3 $$\begin{aligned} v\left( s_{i}\right) =\left( q_{i, 1,1}, \ldots , q_{i, m, 1}, q_{i, 1,2}, \ldots , q_{i, m, 2}, \ldots , q_{i, 1, l}, \ldots , q_{i, m, l}\right) \end{aligned}$$

Then we randomly produce a new vector which is also *Q*-dimensional, denoted by $\phi = (\omega _{1},\ldots , \omega _{Q})$, following the rule in Eq. (4) where each $\omega _{\varphi }$ is randomly produced between -1 and 1. Next, with two *Q*-dimensional vectors $v(s_i)$ and $\phi$, a product operation is adopted in Eq. (5), through which a real value $y_{i}$ is obtained. Furthermore, a sign operation is adopted in Eq. (6) which converts the real value $y_{i}$ into a Boolean one. The reason that we use binary mapping in Eq. (6) is that binary mapping is very time-efficient and effective [27–30], which has been validated and proved by many other literatures.

4 $$\begin{aligned} \omega _{\varphi }=\text {rand}(-1,1)(1 \le \varphi \le Q) \end{aligned}$$

5 $$\begin{aligned} y_{i}=v\left( s_{i}\right) * \Phi =\sum \left( q_{\varphi } * \omega _{\varphi }\right) (1 \le \varphi \le Q) \end{aligned}$$

6 $$\begin{aligned} y_{i}= \left\{ \begin{array}{ll} 1 &{} \mathrm {if}\ y_{i}>0 \\ 0 &{} \mathrm {if}\ y_{i} \le 0 \end{array}\right. \end{aligned}$$

We repeat the above conversion process described in Eqs. (4)-(6) *p* times and then for each student $s_{i}$ $( 1 \le i \le n )$ in matrix *M*, we obtain *p* Boolean values: $y_{i(1)},\ldots , y_{i(p)}$. Thus, a *p*-dimensional vector $Y_{i}$ is obtained as formalized in Eq. (7). In this paper, $Y_{i}$ is the sport index for student $s_{i}$ and we can use $Y_{i}$ to represent $s_{i}$ for further calculation in the subsequent discussions in Step 2 and Step 3. Since $Y_{i}$ is an index containing little sensitive information of student $s_{i}$, the following calculations associated with $Y_{i}$ can be considered privacy-free. This way, the students’ sensitive information contained in historical records can be protected well.

7 $$\begin{aligned} Y_{i}=\left( y_{i(1)}, \ldots , y_{i(p)}\right) \end{aligned}$$

### Step 2: Student clustering

Through Step 1, we have derived the student-index correspondence relationships, i.e., each student $s_{i}$ is corresponding to an index $Y_{i}$. We summarize the above correspondence relationships, i.e., $s_{i} \rightarrow Y_{i}$ with a table shown in Table 1. Here, the hash table is recorded in the cloud platform.

**Table 1:** A hash table

Student	Index
$s_1$	$y_{1(1)},\ldots , y_{1(p)}$
$s_2$	$y_{2(1)},\ldots , y_{2(p)}$
$\ldots$	$\ldots$
$s_n$	$y_{n(1)},\ldots , y_{n(p)}$

To minimize the “false-negative” and “false-positive” probability in subsequent student clustering results, we create *R* hash tables instead of one table, which are presented in Table 2. Thus, each student $s_i$ will be corresponding to *R* indexes: $Index_{1},\ldots , Index_{R}$. Then with Table 2, we can cluster the *n* students in $Stu\_set$ into different groups $G = \{g_{1}, g_{2},\ldots \}$, which is based on the judgment condition in Eqs. (8)-(10). Here, $s_x$, $s_z \in Stu\_set$. Please note that the time complexity of the judgment condition of Eqs. (8)-(10) is very low and therefore, it is typically suitable for the clustering scenarios in big data context since big data processing often involves high computational costs [31–35] and effective computing offloading capabilities [36–40].

$$\begin{aligned} s_{x}\ \text {and}\ s_{z}\ \text {both belong to group}\ g \end{aligned}$$

8 $$\begin{aligned} \text {iff}\ \text {there}\ \text {exists}\ r(r \in [1, R])\ \text {satisfying}\ \left( Y_{x}\right) _{r}=\left( Y_{z}\right) _{r} \end{aligned}$$

9 $$\begin{aligned} (Y_x)_r = y_{x(1)r},\ldots , y_{x(p)r} \end{aligned}$$

10 $$\begin{aligned} (Y_z)_r = y_{z(1)r},\ldots , y_{z(p)r} \end{aligned}$$

**Table 2:** R hash tables

Student	$\boldsymbol I\boldsymbol n\boldsymbol d\boldsymbol e{\boldsymbol x}_{\mathbf1}$	$\boldsymbol I\boldsymbol n\boldsymbol d\boldsymbol e{\boldsymbol x}_{\mathbf2}$	$\boldsymbol\cdots$	$\boldsymbol I\boldsymbol n\boldsymbol d\boldsymbol e{\boldsymbol x}_{\mathbf R}$
$s_1$	$y_{1(1)1},\ldots , y_{1(p)1}$	$y_{1(1)2},\ldots , y_{1(p)2}$	$\cdots$	$y_{1(1)R},\ldots , y_{1(p)R}$
$s_2$	$y_{2(1)1},\ldots , y_{2(p)1}$	$y_{2(1)2},\ldots , y_{2(p)2}$	$\cdots$	$y_{2(1)R},\ldots , y_{2(p)R}$
$\cdots$	$\cdots$	$\cdots$	$\cdots$	$\cdots$
$s_n$	$y_{n(1)1},\ldots , y_{n(p)1}$	$y_{n(1)2},\ldots , y_{n(p)2}$	$\cdots$	$y_{n(1)R},\ldots , y_{n(p)R}$

### Step 3: Sport preference recognition

For two students $s_x$ and $s_z ( \in Stu\_set)$, if they are divided into an identical group $g \in G$, then they are probably with similar sport preferences. In this situation, if $s_z$ likes a sport item $s_i \in SI\_set$, then $s_x$ likes the sport item $s_i$ with high probability, vice versa. This way, we can infer the sport preferences of each student in universities since people belonging to same group often share the same or close preferences with high probability [41–44].

Here, please note that if there are no other students belonging to the group *g* that contains student $s_x$, then an exception occurs since $s_x$ has no similar students. In this situation, we loosen the similar student judgment conditions in Eqs. (8)-(10). Concretely, as Eq. (11) shows, if $s_z$ has the minimal Hamming Distance with $s_x$ in any of the *R* hash tables, then $s_z$ is taken as the similar student of $s_x$ and thus, if $s_z$ likes a sport item $s_i \in SI\_set$, then $s_x$ likes the sport item $s_i$ with high probability, vice versa.

$$\begin{aligned} s_{x}\ \text {and}\ s_{z}\ \text {are}\ \text {similar} \end{aligned}$$

11 $$\begin{aligned} \text {iff}\ (Y_x)_r \oplus (Y_z)_r = \text {MIN}\ {(Y_x)_r \oplus (Y_z)_r | r \in [1, R], s_z \in \ Stu\_set} \end{aligned}$$

In our proposal, each user is assigned an index with less privacy through a hash mapping process, which can be done in an offline manner since the indexes can be generated beforehand [45–47]. Therefore, the time complexity of this conversion process is approximately zero. Afterwards, user clustering can be performed based on an online user index matching process whose time complexity is approximately O(1). Therefore, the total time cost of our proposal is rather low.

Formally, our proposed PESC solution is described with Algorithm 1.

**Algorithm 1:**
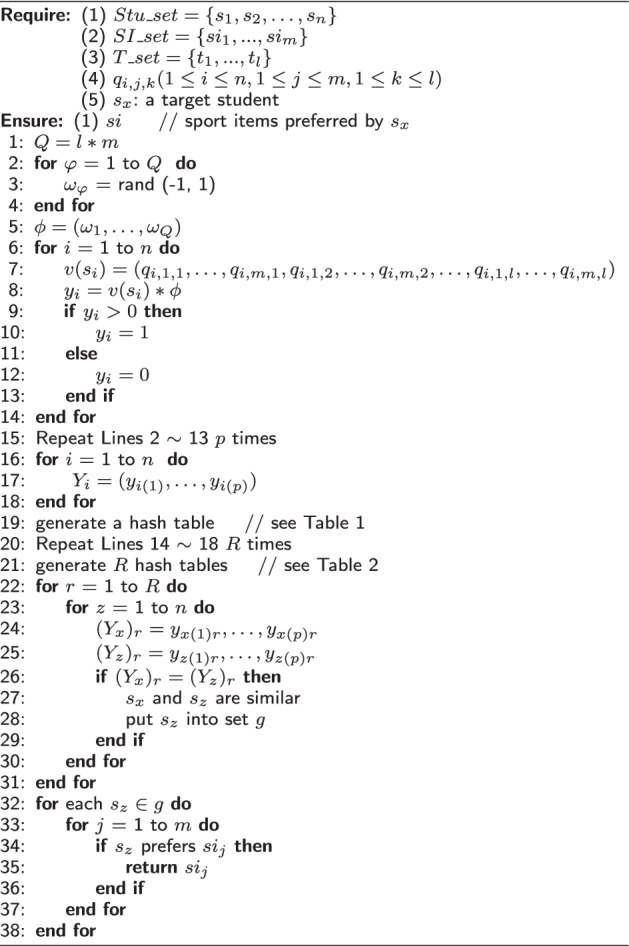
PESC

## Case study

A case study is constructed to show the concrete steps of the proposed PESC solution when we need to cluster students according to their historical sport records in cloud platform and infer their respective sport preferences. Here, we assume there are three students: $s_1$, $s_2$, $s_3$ and each student’s historical sport preferences (totally four sport items) with time (totally two time slots) are presented as follows.

$$\begin{aligned} M= & {} (s_1, s_2, s_3) \\ s_1= & {} \left[ \begin{array}{cccc} 5 &{} 4 &{} 3 &{} 2\\ 4 &{} 3 &{} 2 &{} 1 \end{array}\right] \\ s_2= & {} \left[ \begin{array}{cccc} 4 &{} 5 &{} 3 &{} 4\\ 2 &{} 4 &{} 5 &{} 5 \end{array}\right] \\ s_3= & {} \left[ \begin{array}{cccc} 2 &{} 3 &{} 4 &{} 5\\ 5&3&4&3 \end{array} \right] \end{aligned}$$

Then we convert the three 2*4 matrices corresponding to $s_1$, $s_2$, $s_3$ into three 8-dimensional vectors, i.e., $v(s_1)$, $v(s_2)$, $v(s_3)$. Next, we randomly generate two hash tables, each of which is with three vectors $\phi _1$, $\phi _2$ and $\phi _3$ (as shown in Table 3). The according to Eqs. (4)-(7), we can derive the indexes of the three students $s_1$, $s_2$, $s_3$, i.e., $y_1$, $y_2$, $y_3$ in the two hash tables, respectively. Since $y_1$ = $y_3$ holds in the second hash table (i.e., $y_1$ = $y_3$ = (0, 0, 1)), student $s_3$ is similar with $s_1$ with high probability. Therefore, the sport items preferred by $s_3$ is also preferred by $s_1$ with high probability. This way, we can achieve privacy-aware and efficient student clustering and sport preference inference for sport training in universities.

$$\begin{aligned} v(s_1)= & {} (5, 4, 3, 2, 4, 3, 2, 1)\\ v(s_2)= & {} (4, 5, 3, 4, 2, 4, 5, 5)\\ v(s_3)= & {} (2, 3, 4, 5, 5, 3, 4, 3) \end{aligned}$$

**Table 3:** An example of PESC

Random vector	Hash table 1	Hash table 2
$\phi _1$	(0.1, 0.4, -0.5, 0.3, -0.8, -0.6, 0.4, 0.3)	(-0.1, 0.5, -0.3, 0.5, -0.7, -0.6, 0.2, 0.2)
$\phi _2$	(-0.7, 0.2, 0.4, -0.3, 0.5, 0.2, -0.9, -0.5)	(-0.8, 0.3, -0.5, 0.2, -0.9, -0.6, 0.4, 0.1)
$\phi _3$	(-0.2, 0.8, -0.6, 0.7, -0.3, -0.9, 0.5, 0.2)	(0.6, 0.3, -0.7, 0.8, -0.1, -0.3, 0.7, 0.3)
$y_1 = v(s_1) * \phi$	(-2.4, -1.5, -0.9)	(-2.4, -8.4, 3.4)
$y_2 = v(s_2) * \phi$	(1.6, -7, 3.5)	(1.4, -4.1, 8.6)
$y_3 = v(s_3) * \phi$	(-2.4, -2.7, 1.5)	(-1.3, -6.1, 6.6)
$y_1$	(0, 0, 0)	**(0, 0, 1)**
$y_2$	(1, 0, 1)	(1, 0, 1)
$y_3$	(0, 0, 1)	**(0, 0, 1)**

## Evaluation

Through a time-aware quality performance dataset WS-DREAM, we design a set of experiments to show the effectiveness and efficiency of the PESC solution. We compare the performances (i.e., MAE for measuring clustering accuracy and time cost for measuring clustering efficiency) of PESC with another two existing solutions: UCF (user-based collaborative filtering) and ICF (item-based collaborative filtering). Concretely, we measure the performances of three solutions under different parameter settings. Here, two parameters are recruited: number of students varied from 100 to 300 and matrix density varied from 5% to 40%. Experiments are run in a laptop with 3.20 GHz CPU and 4.0 GB RAM.

Concrete experiment results are reported in the following four profiles.

1. MAE comparison

**Fig. 3:**
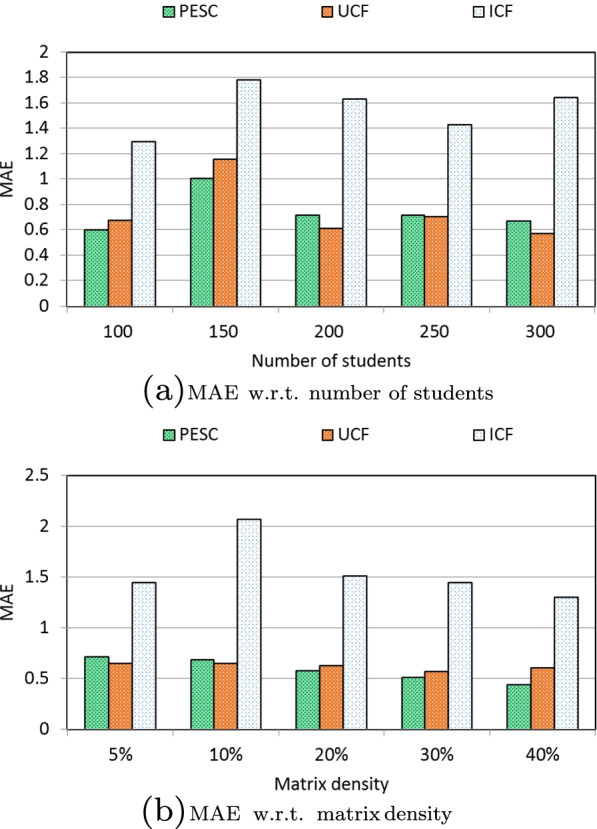
MAE comparisons

We measure the clustering accuracy of three solutions via MAE performances [48–51] under different parameter settings. In concrete, the MAE of three solutions with respect to the number of students is presented in Fig. 3a. As Fig. 3a indicates, the MAE of ICF solution is the highest, which means that the clustering accuracy of ICF is often poor. On the contrary, PESC and UCF solutions both perform well in MAE, which indicates that their clustering accuracy is often high. Furthermore, the MAE value of PESC is close to that of UCF. This means that PESC can achieve approximate clustering accuracy with the baseline UCF solution, because the hash index technique adopted in PESC can guarantee to output the most similar students since the has index technique formalized in Eqs. (4)-(11) is with a good property of similarity keeping. Therefore, our PESC performs well in terms of clustering accuracy.

Moreover, the MAE of three solutions with respect to matrix density is presented in Fig. 3b. As Fig. 3b shows, the MAE of ICF solution is also the highest, which indicates a poor clustering accuracy. On the contrary, PESC and UCF solutions both perform well in MAE, which indicates that their clustering accuracy is often high. Furthermore, like Fig. 3a, the MAE value of PESC is close to that of UCF, which indicates an approximate clustering accuracy between our PESC and baseline UCF. The reason is the same as that analyzed in Fig. 3a, which will not be repeated here.

(2) Time cost comparison

**Fig. 4:**
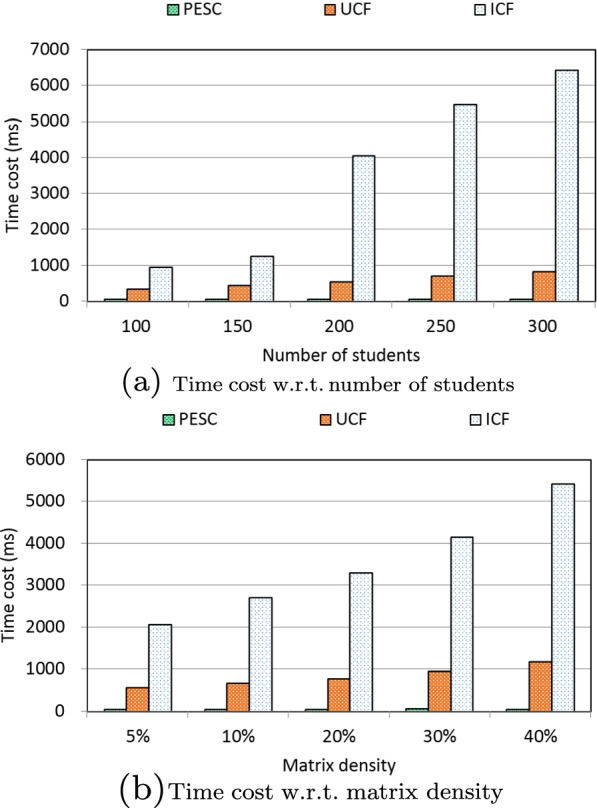
Time cost comparisons

Time cost is a key metric to indicate the algorithm performance especially in the big data environment [52–56]. Here, we measure the clustering efficiency of three solutions via time cost metric under different parameter settings. In concrete, the clustering efficiency of three solutions with respect to the number of students is presented in Fig. 4a. As Fig. 4a indicates, the time cost of ICF solution is the highest since more computational cost is needed for ICF when the number of students is increasing. In addition, the time cost of UCF solution is smaller than ICF, which is still high since all the students need to take part in the similarity calculation. Compared to UCF and ICF, our proposed PESC performs the best in terms of time cost since the hash index technique recruited in PESC is quite efficient due to its low complexity of O(1). Another observation from Fig. 4a is that the time cost of UCF and ICF both increases with the growth of the number of students; while the time cost of our PESC stays approximately stable with the increment of student volume, which indicates a good scalability of PESC in coping with big data.

In addition, the clustering efficiency of three solutions with respect to matrix density is presented in Fig. 4b. Figure 4b show a close result with that of Fig. 4a: the time cost of PESC performs better than UCF and ICF; the time cost of UCF and ICF both increases with the rising of matrix density, while the time cost of PESC stays approximately stable with the growth of matrix density. The reason is the same as that analyzed in Fig. 4a, which will not be repeated here.

(3) MAE of PESC w.r.t. number of hash functions

**Fig. 5:**
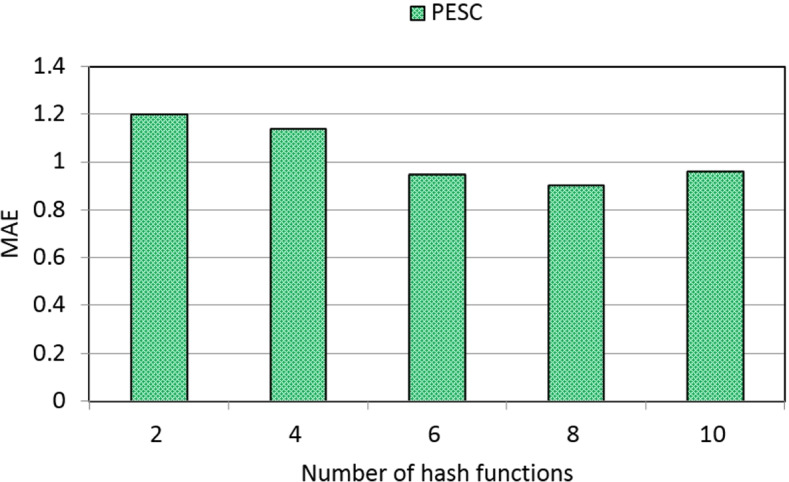
MAE of PESC

In this profile, we measure the clustering accuracy of PESC via MAE metric under different parameter settings. Here, the parameter is the number of hash functions. Measurement results are presented in Fig. 5. As can be seen from Fig. 5, the MAE of PESC approximately decreases with the growth of the number of hash functions. This result can be explained by the inherent property of the hash index technique adopted in PESC. In concrete, when more hash functions are used to generate the hash indexes of students according to Eqs. (4)-(7), the clustering conditions are very strict and therefore, only a smaller number of really similar students are clustered into a same group. In this situation, the clustering accuracy is improved and MAE is decreased accordingly.

(4) Time cost of PESC w.r.t. number of hash functions

**Fig. 6:**
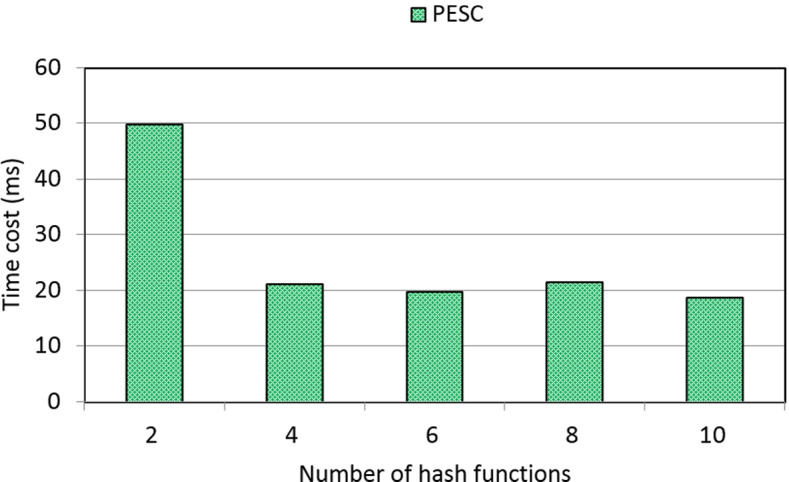
Time cost of PESC

In this profile, we measure the clustering efficiency of PESC under different parameter settings. Here, the parameter is the number of hash functions. Concrete results are presented in Fig. 6. As Fig. 6 indicates, the time cost of PESC first decreases with the growth of the number of hash functions and then stays approximately stable with the growth of the number of hash functions. This result can also be explained by the property of the hash index technique adopted in PESC. Concretely, when more hash functions (e.g., from 2 functions to 4 functions) are recruited in the generation of hash indexes of students according to Eqs. (4)-(7), the clustering conditions become more strict and therefore, only a smaller number of really similar students are clustered into a same group. In this situation, the clustering efficiency is improved significantly. Furthermore, when the number of hash functions continue to become larger (e.g., from 4 functions to 10 functions), the students belonging to same clusters stay stable even the filtering conditions are becoming stricter. In this situation, the time cost also stays approximately stable.

## Conclusions

With the wide adoption of health and sport concepts in human society, how to effectively analyze the personalized sports preferences of each student has become a crucial and emergent task in education. In this situation, past sports training records of students recorded in a central cloud platform have provided a theoretically feasible evaluation basis to cluster the students and then infer their respective sports preferences accordingly. However, the past sports training records of students stored in the cloud platform are often accumulated for years and therefore, the data volume is often large, which often leads to a long processing time of cloud platform for student clustering and sports preference identification. In addition, the past sports training records of students registered in the cloud platform often contain some sensitive user information, which probably discloses user privacy if we cannot protect the data well. Considering these two challenges, a privacy-aware and efficient student clustering approach, i.e., PESC is proposed for sport training cloud-assisted smart education. In concrete, we use hash mapping operations to secure the sensitive user privacy. Firstly, we convert the sensitive user data into a less-sensitive user index through a kind of hash mapping process. Secondly, we use the less-sensitive user indexes to cluster users into different groups without disclosing much user privacy. This way, we can guarantee the user privacy is secure during the accurate user clustering process. We demonstrate the effectiveness of PESC through an intuitive example and a set of experiments.

However, there are some classic privacy protection solutions besides hash adopted in this paper, such as encryption, anonymization, differential privacy, etc [57–59]. In the future work, we will further compare PESC with other privacy-preserving techniques through experiment comparison. In addition, for practical sport-related applicable scenarios, multiple dimensions as well as their weights are meaningful and crucial. Therefore, we will refine PESC by considering weight information of different sport dimensions involved in student clustering scenarios. At last, we only consider one kind of user data (i.e., score) for simplicity in user clustering, while neglecting the diversity of user data [60–63]. In the future work, we will take the data type diversity into consideration to make our research more robust and applicable.

## Data Availability

The WS-DREAM dataset: https://wsdream.github.io/

## Abbreviations

PESC: Privacy-aware and efficient student clustering approach;
SDCM-AA: Sports Drug Control model of Adolescent athletes;
UCF: User-based Collaborative Filtering;
ICF: Item-based Collaborative Filtering.
